# [^64^Cu]NOTA-pentixather enables high resolution PET imaging of CXCR4 expression in a preclinical lymphoma model

**DOI:** 10.1186/s41181-016-0020-6

**Published:** 2017-01-19

**Authors:** Andreas Poschenrieder, Margret Schottelius, Theresa Osl, Markus Schwaiger, Hans-Jürgen Wester

**Affiliations:** 1 Pharmaceutical Radiochemistry, Technische Universität München, Walther-Meißner-Str.3, 85748 Garching, Germany; 2 Department of Nuclear Medicine, Klinikum rechts der Isar, Technische Universität München, Ismaningerstr. 22, 81675 Munich, Germany

**Keywords:** ^64^Cu, Cancer, CXCR4, GPCR, NOTA, Pentapeptide, PET, Radiopharmaceutical, Theranostic, Tracer

## Abstract

**Background:**

The chemokine receptor 4 (CXCR4) is an important molecular target for both visualization and therapy of tumors. The aim of the present study was the synthesis and preclinical evaluation of a ^64^Cu-labeled, CXCR4-targeting peptide for positron emission tomography (PET) imaging of CXCR4 expression in vivo.

**Methods:**

For this purpose, 1,4,7-triazacyclononane,1-glutaric acid-4,7-acetic acid (NODAGA), or 1,4,7-triazacyclononane-triacetic acid (NOTA) was conjugated to the highly affine CXCR4-targeting pentixather scaffold. Affinities were determined using Jurkat T-lymphocytes in competitive binding assays employing [^125^I]FC131 as the radioligand. Internalization and efflux studies of [^64^Cu]NOTA-pentixather were performed in chem-1 cells, stably transfected with hCXCR4. The stability of the tracer was evaluated in vitro and in vivo*.* Small-animal PET and biodistribution studies at different time points were performed in Daudi lymphoma-bearing severe combined immunodeficiency (SCID) mice.

**Results:**

[^64^Cu]NOTA-pentixather was rapidly radiolabeled at 60 °C with high radiochemical yields ≥90% and purities >99%. [^64^Cu]NOTA-pentixather offered the highest affinity of the evaluated peptides in this study (IC_50_ = 14.9 ± 2.1 nM), showed efficient CXCR4-targeting in vitro and was stable in blood and urine with high resistance to transchelation in ethylenediaminetetraacetic acid (EDTA) challenge studies. Due to the enhanced lipophilicity of [^64^Cu]NOTA-pentixather (logP = -1.2), biodistribution studies showed some nonspecific accumulation in the liver and intestines. However, tumor accumulation (13.1 ± 1.5% ID/g, 1.5 h p.i.) was CXCR4-specific and higher than in all other organs and resulted in high resolution delineation of Daudi tumors in PET/CT images in vivo.

**Conclusions:**

[^64^Cu]NOTA-pentixather was fast and efficiently radiolabeled, showed effective CXCR4-targeting, high stability in vitro and in vivo and resulted in high resolution PET/CT images accompanied with a suitable biodistribution profile, making [^64^Cu]NOTA-pentixather a promising tracer for future application in humans.

## Background

Physiologically, the chemokine receptor 4 (CXCR4) and its only endogenous ligand CXCL12 act through G-protein signaling which leads to chemotaxis, cell adhesion, survival, and proliferation (Kuil et al. 2012a; Hattermann & Mentlein 2013; Burger & Kipps 2006). In cancer, the CXCR4-CXCL12 axis is involved in tumor growth and progression, invasion and organ-specific metastasis, therapy resistance as well as recurrence (Chatterjee et al. 2014; Burger & Peled 2009; Domanska et al. 2013). Therefore, CXCR4 is an attractive molecular target, both for therapeutic interventions and for noninvasive quantification of CXCR4 expression, the latter providing important information on the stage and kinetics of the disease. In recent years, a variety of high-affinity CXCR4-targeted imaging probes have been developed for this application, with a focus on targeted peptides, including derivatives of T140 labeled with various radionuclides (George et al. 2014; Yan et al. 2015; Jacobson et al. 2010; Tamamura et al. 2003; Tamamura et al. 1998; Jacobson et al. 2012; Hanaoka et al. 2006), FC131 (Gourni et al. 2011; Demmer et al. 2011a; Demmer et al. 2011b; Tanaka et al. 2010; Poschenrieder et al. 2016a), and small molecules such as AMD3100 (plerixafor)-based derivatives, that have been labeled with ^64^Cu (Weiss et al. 2012; De Silva et al. 2011; Nimmagadda et al. 2010; Jacobson et al. 2009; Woodard et al. 2014), ^18^F (Oltmanns et al. 2011), ^11^C (Hartimath et al. 2014), and ^68^Ga (Poty et al. 2016). Summaries of CXCR4-targeting probes have recently been reported in excellent reviews (Kuil et al. 2012b; Debnath et al. 2013; Weiss & Jacobson 2013). Amongst CXCR4-targeted imaging agents, [^68^Ga]pentixafor (Gourni et al. 2011; Demmer et al. 2011a) holds a prominent position, because its excellent CXCR4-targeting properties and fast renal excretion allow for high contrast PET imaging of CXCR4 expression in humans. Combined with a favorable dosimetry (Herrmann et al. 2015) these properties have paved the way for first currently ongoing clinical studies (Philipp-Abbrederis et al. 2015; Wester et al. 2015; Lapa et al. 2016a; Lapa et al. 2016b; Vag et al. 2016; Herhaus et al. 2016).

Recently, a corresponding therapeutic analog with a slightly modified peptide backbone, [^177^Lu]pentixather, has been introduced, and in first-in-man studies in patients with multiple myeloma, CXCR4-targeted endoradiotherapy using [^177^Lu]pentixather produced promising metabolic responses (Schottelius et al. 2015; Herrmann et al. 2016). [^177^Lu]pentixather thus complements diagnostic imaging with [^68^Ga]pentixafor to a first CXCR4-directed theranostic concept.

However, due to the sensitivity of the pentixafor scaffold towards even small structural modifications such as radiometal exchange in the DOTA chelator (Poschenrieder et al. 2016b), different dedicated precursors are required for diagnostic and therapeutic applications. In this context, copper radionuclides represent an interesting imaging option. Although ^64^Cu (t_1/2_ = 12.7 h, β^+^ = 19%, E_β_^+^_max_ = 656 keV, β^-^ = 38%, E_β_-_max_ = 578 keV) decays by both β^+^ and β^-^ emission, its low β^+^ energy, which is comparable to that of ^18^F (E_β_ + _max_ = 633 keV), provides high spatial resolution in PET imaging (Williams et al. 2005) and its comparably long half-life is advantageous for performing kinetic PET imaging studies over extended periods of time.

To date, only a few ^64^Cu-labeled CXCR4-ligands have been reported. They are either based on the bicyclam AMD3100 (Weiss et al. 2012; De Silva et al. 2011; Nimmagadda et al. 2010; Jacobson et al. 2009) or chelator-conjugated T140 analogs (Jacobson et al. 2012; Jacobson et al. 2011). Despite promising in vitro CXCR4 targeting properties, the biodistribution of these compounds is invariably characterized by high non-specific accumulation and retention of ^64^Cu activity in the excretion organs (20-40% ID/g), especially in liver and kidneys, hinting -at least partly- towards a limited in vivo stability of the respective ^64^Cu-complexes.

Thus, to be able to nevertheless exploit the favorable radionuclide characteristics of ^64^Cu, this study was aimed at developing a ^64^Cu-labeled CXCR4-targeted probe, which combines high ^64^Cu-complex stability with the favorable in vivo pharmacokinetics of pentixafor-based ligands. Thermodynamically stable (Bevilacqua et al. 1987; Wu et al. 2016; Jones-Wilson et al. 1998) and, depending on the conjugated biomolecule, kinetically inert (Zarschler et al. 2014; Dearling et al. 2011) ^64^Cu complexes are formed with a variety of chelators (Cai & Anderson 2014; Wadas et al. 2007), including NOTA.

**Fig. 1:**
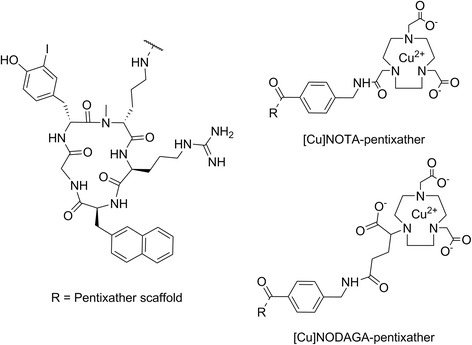
Chemical structures of [Cu]NOTA and [Cu]NODAGA-pentixather

As first demonstrated for [^177^Lu]pentixather, the pentixather scaffold is substantially less sensitive towards structural modifications than pentixafor (Schottelius et al. 2015; Herrmann et al. 2016). Since NOTA forms stable Cu^2+^-complexes and a NOTA-for-DOTA exchange retained the favorable CXCR4-targeting properties of pentixather conjugates (Poschenrieder et al. 2016a), we were interested in the corresponding ^64^Cu-conjugates for PET imaging of CXCR4 expression in vivo. The possibility to further extend this tracer concept towards radiolabeling with copper radionuclides was now exemplarily evaluated by the detailed in vitro and in vivo investigation of ^64^Cu-labeled NOTA- and NODAGA-analogs of pentixather (Fig. 1).

## Results

### Radiolabeling

Under standard labeling conditions, [^64^Cu]NOTA-pentixather was obtained in radiochemical yields ≥90%. Radiochemical purities after C8-light purification were >99%, as confirmed by radio-TLC. The specific activity was 43 GBq/μmol.

### Determination of the lipophilicity (logP)

[^64^Cu]NOTA-pentixather shows a logP of -1.2. Compared to its close structural analog [^18^F]AlF-NOTA-pentixather (log P = -1.4, (Poschenrieder et al. 2016a)), the ^64^Cu-labeled NOTA-peptide is slightly more lipophilic, whereas its lipophilicity is increased by more than an order of magnitude compared to [^68^Ga]pentixafor (log P = -2.90 (Gourni et al. 2011)).

### Determination of CXCR4 affinities (IC_50_)

The CXCR4 affinities of [^nat^Cu]NOTA- and [^nat^Cu]NODAGA-pentixather as well as of the corresponding metal free chelator-conjugated peptides were determined in a competitive binding assay using standard conditions (CXCR4-expressing Jurkat T-lymphocytes (4 × 10^5^ cells per sample) and [^125^I]FC131 as the radioligand (Poschenrieder et al. 2016a; Wester et al. 2015; Schottelius et al. 2015; Poschenrieder et al. 2016b)) (Table 1). Data for [^nat^Ga]pentixafor and FC131 are included as a reference. As already observed for [^nat^Ga]pentixafor, metal complexation leads to enhanced CXCR4 affinity, both for [^nat^Cu]NOTA-pentixather and for [^nat^Cu]NODAGA-pentixather. Compared to [^nat^Ga]pentixafor, however, [^nat^Cu]NOTA-pentixather showed almost twofold higher CXCR4 affinity, while it is reduced by a factor of two in the case of [^nat^Cu]NODAGA-pentixather.

**Table 1:** Affinities (IC_50_) of different Cu^2+^-labeled pentixafor derivatives, [^nat^Cu]NOTA, [^nat^Cu]NODAGA-pentixather, and the corresponding metal-free precursors to human CXCR4. FC131 and [^68^Ga]pentixafor are included as reference compounds. Competitive binding studies were carried out using Jurkat T-cell leukemia cells (400.000 cells/sample) and [^125^I]FC131 as the radioligand

Compound	IC_50_ [nM]
FC131	9.9 ± 2.4^a^
[^nat^Ga]pentixafor (DOTA)	24.8 ± 2.5^a^
[^nat^Cu]DOTAGA-pentixafor	1165 ± 220^a^
[^nat^Cu]NOTA-pentixafor	46.1 ± 26^a^
[^nat^Cu]NODAGA-pentixafor	343 ± 10^a^
[^nat^Cu]DTPA-pentixafor	175 ± 66^a^
NOTA-pentixather	27.5 ± 2.1
[^nat^Cu]NOTA-pentixather	14.9 ± 2.1
NODAGA-pentixather	64.9 ± 6.5
[^nat^Cu]NODAGA-pentixather	47.5 ± 1.8

^a^ref (Poschenrieder et al. 2016b)

Data are means ± SD from a minimum of three separate determinations

### Cell uptake and efflux studies

**Fig. 2:**
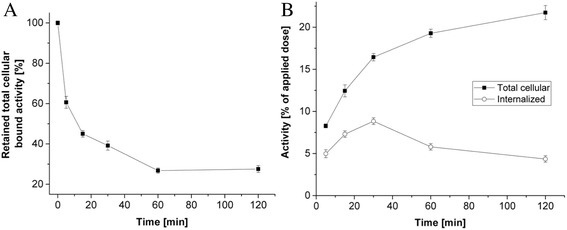
**a** Total cellular uptake and internalization and **b** efflux kinetics of [^64^Cu]NOTA-pentixather in Chem-1 cells stably transfected with hCXCR4; 150000 cells/well were incubated with [^64^Cu]NOTA-pentixather (1 nM) at 37 °C in medium (5% BSA). The total cellular activity was corrected for non-specific binding in the presence of 100 μM AMD3100. Data are means ± SD

Figure 2a shows the CXCR4-specific total cellular uptake and internalization of [^64^Cu]NOTA-pentixather into Chem-1 cells, stably transfected with hCXCR4. The preferential use of this adherent cell line for the internalization and externalization studies was based on our previous observation that the determination of cellular uptake kinetics in suspension cells (such as Jurkat or Daudi cells (Wester et al. 2015)) is oftentimes biased by the experimental conditions (repeated centrifugation and resuspension steps), challenging cell viability and thus leading to inconsistent and irreproducible results. This effect was not encountered using Chem-1 cells. As shown in Fig. 2a, the total cellular [^64^Cu]NOTA-pentixather activity increases steadily over time, reaching approximately 12 and 22% of the added activity after 15 and 120 min, respectively. Internalization reaches a maximum after 30 min (~10%) and decreases to ~ 5% after 120 min. An exemplary efflux study is shown in Fig. 2b and reveals that 28% of the initial cellular [^64^Cu]NOTA-pentixather activity are retained after 120 min of externalization.

### Biodistribution studies

**Fig. 3:**
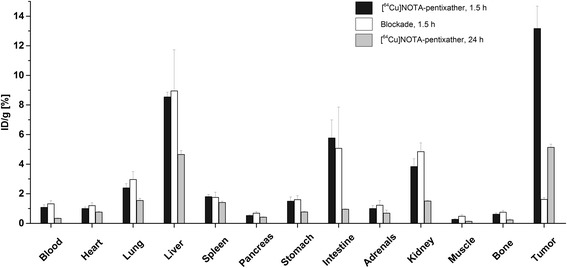
Biodistribution of [^64^Cu]NOTA-pentixather at 1.5 h (tracer only, *black* bars, *n* = 5) and 24 h p.i. (*grey* bars, *n* = 6) in Daudi xenograft-bearing CB-17 SCID mice. Mice were injected with 5.2 MBq [^64^Cu]NOTA-pentixather (122 pmol/0.160 μg peptide; A_S_ = 43 GBq/μmol). Non-specific tracer accumulation was determined by coinjection of 2 mg/kg AMD3100 (50 μg/mouse; *white* bars, *n* = 3)

**Fig. 4:**
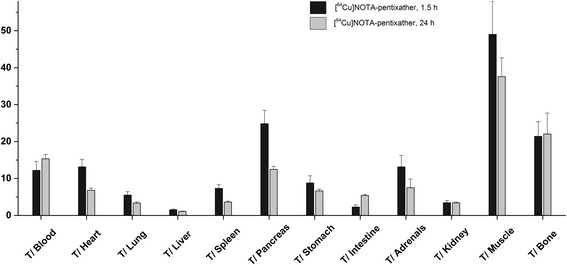
Tumor-to-organ ratios of [^64^Cu]NOTA-pentixather 1.5 and 24 h p.i. (*black* and *grey* bars, respectively)

Biodistribution data of [^64^Cu]NOTA-pentixather at 1.5 h p.i. (*n* = 5, black bars) and 24 h p.i. (*n* = 6, grey bars) in Daudi-lymphoma-bearing SCID mice are summarized in Fig. 3. Given the fact that Jurkat cells, which are generally used for the determination of CXCR4 affinity, are not tumorigenic in mice and to ensure comparability of data with previous studies (Poschenrieder et al. 2016a; Wester et al. 2015; Schottelius et al. 2015), the Daudi lymphoma model was also used in this study. Blood clearance of [^64^Cu]NOTA-pentixather was fast (1.1 ± 0.2% ID/g at 1.5 h p.i.), and activity accumulation in non-target organs was generally low. Due to the enhanced lipophilicity of [^64^Cu]NOTA-pentixather compared to [^68^Ga]pentixafor (logP = -2.90 (Gourni et al. 2011)), a certain extent of hepatobiliary excretion and thus non-specific accumulation in the liver and intestines were observed (7.2 ± 1.1 and 5.0 ± 2.8% ID/g, respectively), while activity accumulation in the kidney was comparably low (3.8 ± 0.5% ID/g). Tumor uptake of [^64^Cu]NOTA-pentixather was significantly higher than tracer accumulation in all other organs (13.1 ± 1.5% ID/g), underlining its excellent CXCR4 targeting efficiency. Coinjection of 2 mg/kg AMD3100 (Fig. 3, white bars) resulted in a reduction of tumor uptake by 88% (1.5 h p.i.), demonstrating the high CXCR4-specificity of [^64^Cu]NOTA-pentixather accumulation in the lymphoma xenograft. The resulting tumor-to-organ ratios at 1.5 and 24 h p.i. are summarized in Fig. 4. While the tumor-to-background ratios for most tissues remain unchanged or decrease within the observation period, t/blood and t/intestine ratios increase 1.3 and 2.4 fold between 1.5 and 24 h p.i., respectively.

### Small animal PET imaging

**Fig. 5:**
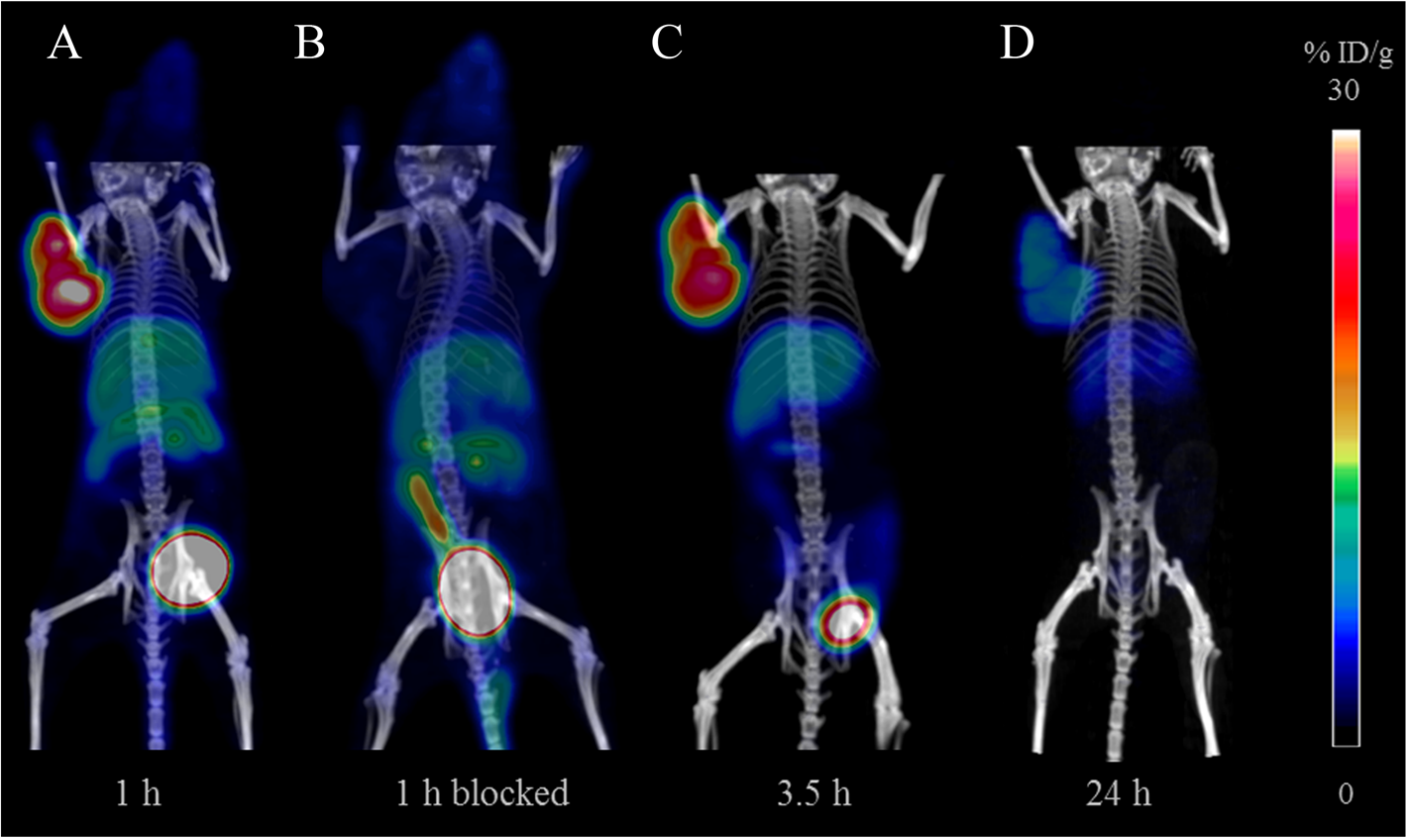
PET/CT imaging (maximal intensity projections (MIP)) of Daudi xenograft bearing CB-17 SCID mice at 1 h p.i. of 5.2 MBq [^64^Cu]NOTA-pentixather; (**a**) tracer only, (**b**) coinjection of 2 mg/kg AMD3100; (**c**) and (**d**) images at 3.5 and 24 h p.i. (tracer only)

Representative PET/CT images of [^64^Cu]NOTA-pentixather in Daudi-lymphoma bearing SCID mice at 1 h, 3.5 h, and 24 h p.i. are shown in Fig. 5. Besides high and CXCR4-specific uptake of [^64^Cu]NOTA-pentixather in the Daudi lymphoma xenograft, some background activity uptake is observed in the liver, the gall bladder, and the intestines. Upon coinjection with 50 μg AMD3100, tumor accumulation is reduced to background levels (Fig. 5b), demonstrating that tumor uptake of [^64^Cu]NOTA-pentixather is almost exclusively CXCR4-mediated.

**Fig. 6:**
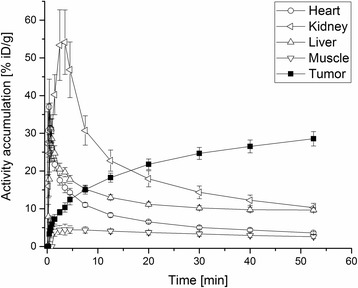
Time-activity-curves of heart, kidney, liver, muscle, and tumor after injection of [^64^Cu]NOTA-pentixather into Daudi lymphoma-bearing SCID mice

Figure 6 shows time-activity-curves (TACs) obtained by [^64^Cu]NOTA-pentixather PET for the heart (blood pool), kidney, liver, muscle, and tumor and further illustrates the rapid and continuous accumulation of [^64^Cu]NOTA-pentixather in the Daudi xenograft, accompanied by rapid background clearance.

### Metabolite analysis and EDTA challenge

**Fig. 7:**
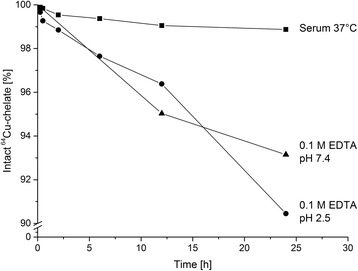
In vitro stability of [^64^Cu]NOTA-pentixather as a function of incubation time. [^64^Cu]NOTA-pentixather was incubated in human serum at 37 °C (■) or 0.1 M EDTA at different pH values (7.4: ▲, 2.5: ●) at 25 °C

To investigate the in vitro stability of [^64^Cu]NOTA-pentixather, the tracer was incubated both in human serum at 37 °C and in 0.1 M EDTA at pH = 2.5 and physiological pH (7.4) at RT for different time points up to 24 h (Fig. 7). [^64^Cu]NOTA-pentixather remained stable over 24 h in human serum (>99% intact ^64^Cu-chelate). Additionally, high resistance towards transchelation was demonstrated in challenge experiments with excess EDTA (0.1 M) (logK_Cu-EDTA_ = 18.7 (Jones-Wilson et al. 1998)), since over 90% of [^64^Cu]NOTA-pentixather were found to remain intact, even at pH 2.5 after 24 h.

The in vivo stability of the tracer was investigated via metabolite studies in mice. Radio-HPLC analysis of urine and blood as well as tissue homogenates from liver and kidney showed that 98% intact [^64^Cu]NOTA-pentixather were present in blood and urine, while 68 and 24% intact tracer were found in liver and kidney, respectively.

## Discussion

Excellent reviews on the multiple facets of copper chelation chemistry highlight the various aspects that need to be taken into account in the development of copper-radiopharmaceuticals with suitable in vivo biodistribution profiles (Cai & Anderson 2014; Wadas et al. 2007; Anderson & Ferdani 2009; Tegoni et al. 2014).

Despite reasonable thermodynamic stability, macrocyclic Cu^2+^ complexes, e.g. DOTA-complexes, are oftentimes prone to in vivo dissociation and transchelation due to challenge with e.g. endogenous metal ions or metal-binding proteins under very dilute tracer concentrations (Jones-Wilson et al. 1998; Kukis et al. 1994), leading to significant non-specific activity accumulation in the liver or other non-target tissues (Bass et al. 2000; Boswell et al. 2004) at least at later time points. Thus, copper complexes with high kinetic inertness, which are formed with specific macrocyclic nonbridged (e.g. cyclen and cyclam derivatives, including 1,4,8,11-tetraazacyclododecane-1,4,8,11-tetraacetic acid (TETA), NOTA or NODAGA), bridged macrocyclic chelators, (e.g. 1,8-ethylene cross-bridged cyclam derivatives like CB-TE2A), or sarcophagine-based chelators (e.g. diamSar) are primarily used for the preparation of copper radiopharmaceuticals (Cai & Anderson 2014; Wadas et al. 2007; Wadas et al. 2010).

Unfortunately, Cu^2+^-complexes of different pentixafor derivatives (Table 1) showed disappointing affinities towards CXCR4. However, due to structural modifications of the pentixafor scaffold, we found a conjugate, [Al^18^F^2+^]NOTA-pentixather, which demonstrated promising CXCR4-targeting properties in vitro and in vivo as well as suitable pharmacokinetics (Poschenrieder et al. 2016a). Thus, NOTA-pentixather as well as the corresponding NODAGA-analog were evaluated in this study as precursors for novel CXCR4-targeted ^64^Cu-radiopharmaceuticals. Because of the comparably low CXCR4 affinity of the [^nat^Cu]NODAGA conjugate (Table 1), only [^nat^Cu]NOTA-pentixather (Fig. 1) was selected for further preclinical evaluation.

Although [^64^Cu]NOTA chelates are generally of high stability (Zarschler et al. 2014; Dearling et al. 2011; Prasanphanich et al. 2007), dissociation of ^64^Cu has been reported to be influenced by the conjugated biomolecule, e.g. by exposed amino acid residues that compete with the chelator (Zarschler et al. 2014; Kukis et al. 1994; Boswell et al. 2004). Stability evaluation of [^64^Cu]NOTA-pentixather in vitro by means of serum incubation, acid-promoted dissociation studies, and EDTA-challenge experiments resulted in almost no transchelation when challenged and no albumin association. In vivo investigations demonstrated ≥98% intact ^64^Cu-chelate in urine and blood, whereas transchelation of ^64^Cu in kidney and liver occurred; Cu-dependent enzymes (e.g. superoxide dismutase (SOD)) or proteins (e.g. caeruloplasmin and metallothionein) are highly abundant in liver or kidney and are most probably responsible for inferior kinetic inertness of the tracer in these organs (Bass et al. 2000; McArdle et al. 1999; Terao & Owen 1973; Blower et al. 1996; Valentine et al. 1999; Musci et al. 1999). Transchelation of [^64^Cu]TETA-octreotide to SOD (Bass et al. 2000) and transfer of copper from a ^67^Cu-labeled antibody to ceruloplasmin (Mirick et al. 1999) were previously reported, accompanied by increasing blood activity levels, indicating the re-release of ^64^Cu from liver to the blood. However, this was not the case for [^64^Cu]NOTA-pentixather, which showed no dissociation in blood, both in vitro and in vivo, and displayed increasing tumor-to-blood ratios over time.

Moreover, activity accumulation in excretory organs 1.5 h p.i. was similar to that of [^18^F]AlF-NOTA-pentixather (logP = -1.4); hence, the shift towards hepatobiliary excretion and thus unspecific accumulation in the liver and intestines can be attributed to its slightly enhanced lipophilicity (logP = -1.2) rather than the instability of the ^64^Cu-chelate. Although NOTA causes a slightly enhanced lipophilicity compared to the parental compound [^68^Ga]pentixafor (logP = -2.9), NOTA offered suitable properties as chelator in novel pentixafor-based ^64^Cu-radiopharmaceuticals.

Although no transchelation or metabolization assays have been performed for the reported ^64^Cu-labeled T140 derivatives (T140-2D, DOTA-, and NOTA-T140-NFB), the authors suggest the formation of metabolites of the labeled peptides in vivo because, in contrast to [^18^F]T140, all of them shared high, long-lasting, and mostly unspecific uptake in liver and kidneys (Jacobson et al. 2012; Jacobson et al. 2011) which impairs their use as ideal PET imaging agent.

Besides the tetradecapeptides, two AMD-based CXCR4-targeting ^64^Cu-labeled tracers have been reported, namely [^64^Cu]AMD3100 and [^64^Cu]AMD3465 (Weiss et al. 2012; De Silva et al. 2011; Nimmagadda et al. 2010; Jacobson et al. 2009). The latter providing very high and specific tumor uptake; however both tracers also show high uptake in the liver and kidneys. In contrast to ^64^Cu-NOTA-pentixather and ^64^Cu-T140 derivatives, uptake in the liver was mostly specific; therefore, the authors suggest a CXCR4-independent component (Jacobson et al. 2009). Moreover, as tested by [^64^Cu]CuCl_2_ injected mice, transchelation did not contribute to the increased uptake in kidneys or liver. However, after 10 h, % ID/g values in the blood increased which hints towards transchelation at delayed time points (De Silva et al. 2011).

In the case of [^64^Cu]NOTA-pentixather, the overall high stability is also reflected by increasing tumor-to-blood ratios, which, accompanied with its promising CXCR4-targeting properties, resulted in high-contrast PET/CT images of lymphoma xenografts (Fig. 5). As shown in TACs, tumor uptake was higher than activity accumulation in all other tissues already at 17 min p.i., highlighting the efficient CXCR4-targeting and fast clearance of the tracer from non-target tissue with comparably low unspecific background accumulation at all time points (Fig. 6). However, at 1.5 and 3.5 h p.i, some background accumulation in the excretion organs was still visible. As discussed, this hepatic and intestinal activity uptake is most probably the result of a slightly delayed overall clearance due to the enhanced lipophilicity of the tracer compared to [^68^Ga]pentixafor. At 24 h p.i., tracer clearance from the body is almost complete, resulting in very low residual activity in liver and tumor. Modest internalization and fast externalization kinetics (Fig. 2) contribute to the fast clearance from tumor tissue. Although, its use for endoradiotherapeutic purposes is therefore limited, [^64^Cu]NOTA-pentixather proved as valuable PET agent for imaging of CXCR4 expression in vivo.

## Conclusion

In summary we were able to successfully transfer our ‘pentixafor/pentixather’-based CXCR4-targeting technology to a promising analog for ^64^Cu-labeling. Due to the suitable in vitro and in vivo stability of the tracer, its rapid and specific activity accumulation in the Daudi xenograft, as well as rapid clearance from the background, PET images resulted in a clear delineation of the experimental tumors in vivo. However, fast clearance from the human xenografts and some uptake in the excretory organs, attributed to its slightly enhanced lipophilicity, limit the tracer’s efficiency for a further therapeutic transfer with ^67^Cu. The initial results recommend further investigations with [^64^Cu]NOTA-pentixather for CXCR4-PET imaging.

## Methods

General procedures and syntheses of the peptides were performed as described (Poschenrieder et al. 2016b).

### [^nat^Cu]NOTA-pentixather

The non-radioactive reference compound [^nat^Cu]NOTA-pentixather was prepared by mixing a 2 mM solution of NOTA-pentixather (250 μL) with an equal volume of 2 mM Cu(OAc)_2_ (pH = 6.0) and allowing the complexation reaction to proceed at room temperature (RT) for 30 min. Quantitative complex formation was confirmed by HPLC analysis.

HPLC (30 to 55%B in 15 min): t_R_ = 12.3 min; calculated monoisotopic mass for [^nat^Cu]NOTA-pentixather (C_56_H_70_CuIN_13_O_12_): 1306.4; found (ESI-MS): m/z = 1307.7 [M + H]^+^.

### [^nat^Cu]NODAGA-pentixather

The non-radioactive reference compound [^nat^Cu]NODAGA-pentixather was prepared as described above. Quantitative complex formation was confirmed by HPLC analysis.

HPLC (30 to 55%B in 15 min): t_R_ = 12.3 min; calculated monoisotopic mass for [^nat^Cu]NOTA-pentixather (C_59_H_74_CuIN_13_O_14_): 1378.4; found (ESI-MS): m/z = 1379.8 [M + H]^+^.

### ^64^Cu-labeling of NOTA-pentixather

[^64^Cu]CuCl_2_ in 0.1 M HCl was obtained from the University of Tübingen. For peptide radiolabeling, 25 μL of [^64^Cu]CuCl_2_ (273 MBq) were added to an aqueous solution containing 150 μL 0.4 M NaOAc buffer (pH = 5.5) and 5 nmol of NOTA-pentixather. After heating to 60 °C for 10 min, the reaction mixture was allowed to cool to RT, and [^64^Cu]NOTA-pentixather was isolated via solid phase extraction using a C8-light cartridge (Waters). The product was eluted from the solid phase using a small volume of ethanol containing 0.5% (v/v) acetic acid.

### EDTA-challenge

For challenging experiments, 4 MBq of [^64^Cu]NOTA-pentixather were incubated in 0.1 M EDTA at different pH values (2.5 and 7.4) for a minimum of 24 h. At specific time points, samples were analyzed via radio-TLC using silica gel impregnated chromatography paper (Agilent Technologies, CA, USA) and 0.1 M phosphate buffer (pH = 7.4) containing 10 mM sodium EDTA as the mobile phase.

### Determination of lipophilicity and serum stability

The lipophilicity and serum stability of [^64^Cu]NOTA-pentixather were determined as described previously (Poschenrieder et al. 2016a). Briefly, to a solution of app. 2 kBq of radiolabelled peptide in 500 μL of PBS (pH 7.4), 500 μL of octanol were added (*n* = 6). Vials were vortexed vigorously for 3 min. To achieve quantitative phase separation, the vials were centrifuged at 14,600 · g for 6 min in a Biofuge 15 (Heraeus Sepatech, Osterode, Germany). The activity concentrations in 100 μL samples of both the aqueous and the organic phase were measured in a gamma counter, and the log P_ow_ was calculated.

### In vitro studies

CXCR4 affinities were determined using Jurkat T-cell leukemia cells and [^125^I]FC131 as the radioligand (Poschenrieder et al. 2016b). Briefly, cells (4 × 10^5^ cells per vial) were incubated with app. 0.1 nM of [^125^I]FC131 in the presence of varying concentrations (10^−11^ to 10^−5^ M) of the respective peptide of interest (n = 3 samples/concentration) for 120 min at RT. After centrifugation and repeated washing steps, the amount of free and bound radioligand were quantified for each sample using a γ-counter, and IC_50_ values were calculated using GraphPad Prism 6.01 software.

Internalization and efflux studies were performed as described (Poschenrieder et al. 2016a) using Chem-1 cells stably transfected with hCXCR4 (HTS004C, Merck Millipore, Darmstadt, Germany). Briefly, adherent cells in 24-well plates (150,000/well) were incubated with 1 nM [^64^Cu]NOTA-pentixather in the absence (total binding) or presence of 100 μM AMD3100 (non-specific binding) at 37 °C for different time intervals up to 60 min. Subsequently, the amount of free, membrane-bound, and internalized activity were quantified for each sample using a γ-counter. For efflux studies, [^64^Cu]NOTA-pentixather was first allowed to internalize at 37 °C for 45 min. Then, the supernatant was exchanged by ligand-free assay medium, and efflux of [^64^Cu]NOTA-pentixather over time was investigated (37 °C). The remaining total cellular activity (membrane-bound + internalized activity) at different time points was quantified as described above.

### In vivo studies

All animal studies were approved by the local authorities (No.: 55.2-1-54-2532-71-13) and are in compliance with the institution’s guidelines. For metabolite analysis, 18 MBq of [^64^Cu]NOTA-pentixather in a total volume of 200 μL of phosphate-buffered saline (PBS) were injected into the tail vein of a CB17 SCID mouse. At 1 h p.i., the animal was sacrificed and blood, urine, kidneys, and liver were collected. After sample preparation (Weineisen et al. 2014), the samples were analyzed by reversed phase (RP)-HPLC.

For PET and biodistribution studies, an average of 5.2 MBq [^64^Cu]NOTA-pentixather (200 μL in PBS, 122 pmol, 160 ng) with a SA of 43 GBq/μmol was injected intravenously into the tail vein of isofluorane anaesthesized female Daudi (human B-cell lymphoma) xenograft bearing CB17 SCID mice. CXCR4-specificity of [^64^Cu]NOTA-pentixather uptake was demonstrated by coinjection of 2 mg/kg AMD3100. PET imaging with [^64^Cu]NOTA-pentixather under ‘tracer only’ (*n* = 2) as well as blocking conditions (coinjection of 2 mg/kg AMD3100, n = 1) was performed at three different time points (dynamic PET imaging for 1 h, followed by static image acquisition for 15 min after 3.5 and 24 h) using an Inveon Siemens μPET scanner. PET/CT images were reconstructed by a two-dimensional ordered subset expectation maximum (2D-OSEM) algorithm with no attenuation correction. Image analysis was performed using the Inveon software, and results were calculated as %ID/g. For biodistribution studies, the mice were sacrificed at 1.5 h (*n* = 5) and 24 h (*n* = 6) p.i., tissues and organs of interest were dissected, weighed, and counted for radioactivity in a γ-counter. The percentage of injected dose per gram of tissue (% ID/g) was calculated; data are presented as mean ± SD.

## Abbreviations

AMB: Aminomethylbenzoyl
A_S_: Specific activity
CXCR4: Chemokine receptor 4
DOTA: 1,4,7,10-tetra azacyclododecane-1,4,7,10-tetraacetic acid
EDTA: Ethylenediaminetetraacetic acid
GRPR: Gastrin-releasing peptide receptor
IC_50_: Half maximal inhibitory concentration
MIP: Maximal intensity projection
NH_4_OAc: Ammonium acetate
NODAGA: 1,4,7-triazacyclononane,1-glutaric acid-4,7-acetic acid
NOTA: 1,4,7-triazacyclononane-triacetic acid
p.i.: Post injection
PBS: Phosphate-buffered saline
PET: Positron emission tomography
RP-HPLC: Reversed phase high-performance liquid chromatography
RT: Room temperature
SCID: Severe combined immunodeficiency
SOD: Superoxide dismutase
TAC: Time-activity curve
TETA: 1,4,8,11-tetraazacyclododecane-1,4,8,11-tetraacetic acid
